# Hydrothermal Aging of ATZ Composites Based on Zirconia Made of Powders with Different Yttria Content

**DOI:** 10.3390/ma14216418

**Published:** 2021-10-26

**Authors:** Marek Grabowy, Agnieszka Wilk, Radosław Lach, Zbigniew Pędzich

**Affiliations:** 1 IEN Institute of Power Engineering, Ceramic Branch CEREL, 8 Mory Street, 01-330 Warsaw, Poland; grabowy@cerel.pl; 2 AGH-Department of Ceramics and Refractories, Faculty of Materials Science and Ceramics, AGH University of Science and Technology, 30 Mickiewicza Avenue, 30-059 Krakow, Poland; radoslaw.lach@agh.edu.pl (R.L.); pedzich@agh.edu.pl (Z.P.)

**Keywords:** alumina toughened zirconia, composites, low temperature degradation, hydrothermal aging

## Abstract

The presented work concerns the development and investigation of three different grades of ZrO_2_ materials containing Al_2_O_3_ particles (ATZ-Alumina Toughened Zirconia ceramics with 2.3–20 vol.% of alumina). The zirconia powders containing 3 mol.% of yttria were synthesized by a precipitation/calcination method and fabricated from two different zirconia powders with different yttria content. Then, the selected ATZ composites (ATZ-B, ATZ-10 and ATZ-20) were prepared by means of conventional mixing, compacting and sintering at 1450 °C for 1.5 h. The phase composition, microstructure, relative density and basic mechanical properties were determined. Uniform microstructures with relative densities over 99% of theoretical density, hardness values between 12.0–13.8 GPa, flexural strength up to 1 GPa and outstanding fracture toughness of 12.7 MPa⋅m^1/2^ were obtained. The aging susceptibility of alumina toughened zirconia materials, as a consequence of hydrothermal treatment, was investigated. The aim of this study was to determine the influence of LTD (low temperature degradation) on the tetragonal to monoclinic phase transitions and on the flexural strength of hydrothermally aged specimens. The results were compared to those obtained for commercially available tetragonal zirconia-based materials containing 3 mol.% of yttria. This research shows that ATZ composites that have excellent mechanical properties and sufficient hydrothermal aging resistance can be attained and later used in technical and biomedical applications.

## 1. Introduction

The system of zirconia (ZrO_2_)-alumina (Al_2_O_3_) is gaining increasing attention due to its inherent intrinsic properties and hence its applicability in structural and biomedical ceramics [1,2,3,4,5,6]. In the ZrO_2_-Al_2_O_3_ system, ZrO_2_ reinforced with Al_2_O_3_ particles, called ATZ (alumina toughened zirconia), was found to be more effective in enhancing the mechanical properties when compared to its monoclinic counterparts [7,8]. Alumina ceramics have outstanding elastic modulus, hardness, wear properties and high chemical stability under corrosion conditions. Tetragonal zirconia-based ceramics show excellent toughness and high bending strength, properties that are obtained because of undergoing a tetragonal to monoclinic (t–m) phase transformation that increases the mechanical properties of these materials. The stabilization of the metastable tetragonal and cubic structures can be achieved by doping zirconia with oxygen vacancies only. There is critical minimum and maximum oxygen vacancy concentration which, when exceeded, results in a phase transformation. Moreover, both counterparts are characterized by excellent biocompatibility. The use of the synergistic effect of improving the mechanical properties in ATZ composites allows the significant increase in the fracture toughness (11 MPa⋅m^1/2^) and flexular strength (>1.5 GPa) of these ceramics, which also strictly depends on the phase composition and obtained microstructure [9,10].

However, 3 mol.% yttria-tetragonal zirconia ceramics are sensitive to humid conditions and undergo a spontaneous (without being subjected to stress) tetragonal to monoclinic phase transformation in the presence of water or water vapors at relatively low temperatures (from room temperature to about 400 °C) [11,12]. This phenomenon is called low temperature degradation (LTD, also referred to as hydrothermal aging or hydrothermal degradation). The LTD phenomenon strongly depends on the grain size and surface residual stress [13,14]. The aging of the zirconia is a very serious problem for dentistry [15] and the orthopedic community [3], where zirconia surfaces are directly exposed to intrabody hydrothermal environments. It also causes many difficulties in industries where contact with water is unavoidable and long-term performance is required, for example in solid-oxide fuel cells or thermal barrier coatings [16,17].

There are several theoretical mechanisms that have been proposed to explain the course of the LTD phenomenon. According to Sato et al. [12] a reaction between water and Zr-O-Zr bonds occurs at surface flaws, which results in Zr-OH formation and destabilization of the tetragonal phase. The t-m transformation starts at the surface and propagates inside the material. Hughes et al. [18] suggested the annihilation of surface oxygen vacancies by generation of O^2−^ ions, which then penetrate into the lattice and promote t-m phase transformation. Guo et al. [19] proposed a mechanism consisting of several steps: water adsorption on ZrO_2_ surface, a reaction between H_2_O with O^2−^ and ZrO_2_, formation of Zr-OH with hydroxyl groups, penetration of OH^−^ anions (they migrate faster than O^2−^ because of their similar size and lesser electric charge) into the interior by grain boundary diffusion, filling of oxygen vacancies by hydroxyl groups combined with their annihilation and formation of protonic defects, reduction of oxygen vacancy concentration, destabilization of tetragonal phase and occurrence of t-m structure transition. Moreover, Guo et al. [19] stated that over a wide range of elevated vapor pressure, water molecules could be incorporated into the tetragonal ZrO_2_ lattice. Water molecules fill the oxygen vacancies according to following equation: $H_{2}O+V_{0}^{••}+O_{0\;}^{x}\underset{}{\Leftrightarrow }2{\left(OH\right)}_{0}^{•}$. This induced ${\left(OH\right)}_{0}^{•}$ protonic defects and, as a result, the tetragonal structure transforms to the monoclinic one. Moreover, according to Lange et al. [20], this protonic defect ${\left(OH\right)}_{0}^{•}$ reacts with $Y_{Zr}^{\prime }$ ions and forms Y(OH)_3_. The presence of Y(OH)_3_ crystallite clusters was confirmed by TEM- Transmission Electron Microscopy observations in hydrothermally aged, Y_2_O_3_-stabilized ZrO_2_ ceramics. As a consequence, it comes to the depletion of stabilizers from the grains and spontaneous t-m transformation. The t-m phase transition results in crack formation, surface roughening (owing to volume expansion) and severe degradation of mechanical properties.

It is necessary to mention that the degradation behavior of 3Y-TZP is expected to be changed when different dopants or additives are applied [21]. The addition of Al_2_O_3_, which is not susceptible to hydrothermal aging, is a very promising way to increase the reliability and stability of zirconia materials [22,23,24,25].

However, there is a gap in the literature regarding the corrosion resistance of ATZ composites under varying conditions, especially over an extended period of time at temperatures >150 °C, which does not give the answer to the crucial question about aging. Based on the growing interest in the use of ATZ ceramic materials, the aim of the presented work is to determine the mechanical properties (such as hardness, elastic modulus and fracture toughness) in the selected ATZ composites, in comparison to commercial zirconia powders based ceramics containing 3 mol.% of yttria. The main goal of the study is to investigate the corrosion of ATZ composites in water under hydrothermal conditions and provide an assessment of its influence on basic characteristics, such as flexural strength and phase composition (t-m transformation).

## 2. Materials and Methods

The starting powder, 3 mol.% yttria-tetragonal zirconia, was prepared by a precipitation/calcination method utilizing two zirconia powders—the first one was powder of pure nanometric ZrO_2_ and the second one was a solid solution of 4 mol.% Y_2_O_3_ in ZrO_2_, as detailed elsewhere [26]. The powder of the base material (hereinafter referred to as ATZ-B) was obtained by adding a small amount (2.3 vol.%) of nanometric alumina powder (TM-DAR, Taimicron, Taimei Chemicals Co. LTD., Tokyo, Japan) to the starting zirconia powder. Moreover, based on the ATZ-B material, two ATZ composites containing a greater addition of corundum were also prepared. They are abbreviated as ATZ-10 and ATZ-20 and in their final composition they contained 10 vol.% and 20 vol.% of nanometric alumina, respectively. ATZ composites were prepared by blending appropriate quantities of zirconia and alumina powder by attritor milling using zirconia milling media (3 mm in diameter) and isopropyl alcohol as mixing agent. The mixtures were dried overnight. The composite powders were compacted under a uniaxial pressure of 50 MPa in a hydraulic press (Specac) into disc samples and subsequently cold isostatic pressed at 200 MPa. Green samples were conventionally sintered in an air atmosphere, in the presence of zirconia powder bed at 1450 °C with a holding time at the maximum temperature of 1.5 h, using very slow heating and cooling rate. At the same time, the reference samples were prepared in an identical manner, made of a commercially available zirconium oxide powder containing 3 mol.% of yttrium oxide (3Y-TZP, TOSOH Co. LTD., Tokyo, Japan). This reference material was designated in the text as 3Y-TZP.

The bulk densities of the sintered bodies were measured according to the Archimedes principle in water. Their theoretical densities were calculated according to the rule of mixtures by taking into account the theoretical densities of the ATZ individual components. Prior to the hardness measurements and microscopic observations, the samples were first hand- and later machine-polished using Diamond Pads (Struers, Cleveland, OH, USA) and MD-Polishing Cloth (Struers, Cleveland, OH, USA) with 1 µm diamond paste to obtain a mirror-like surface. The hardness and K_IC_ were examined using the Vickers indentation technique. The indentation load (applied for 10 s) was kept at 9.81 N for HV - Vickers Hardness and at 49.05 N for K_IC_ measurements. The K_IC_ was calculated according to the Niihara equation [27] using the length of the Palmquist cracks and indentations’ sizes. Average results were obtained from at least five measurements for every sample. The Young’s modulus was investigated by the method of ultrasonic wave velocity measurements (UZP-1, Inco-Veritas, Warsaw, Poland). Microstructures of the ATZ composites were examined on polished and thermally etched (1200 °C, 1 h) surfaces using scanning electron microscopy (Nova NanoSEM 200, FEI - Field Electron and Ion Company, Hillsboro, OR, USA). The obtained images were manually binarized with the Procreate application (version 5.1.8, Savage Interactive, North Hobart, Tasmania, Australia) and then analyzed using ImageJ (version 1.53j, Wayne Rasband, Kensington, MD, USA). Sufficiently accurate average results from measurements of at least 1000 grains were reported.

All ATZ composites (ATZ-B, ATZ-10 and ATZ-20) and the reference material were subjected to hydrothermal aging (Low Temperature Degradation, LTD). The samples were placed in a Teflon container and then to a steam autoclave (Parr Instrument Company, Moline, IL, USA) at water vapour conditions using different aging temperatures and times. The autoclaving conditions are presented in Table 1. Hydrothermal aging stability was assessed by determining the phase transformation (with XRD- X-Ray Diffraction, X’Pert Pro, EMPYREAN system, PANalytical, Malvern, United Kingdom) and flexural strength changes after aging treatments.

The phase compositions for both starting and aged ATZ composites were analyzed by X-ray diffractometer (X’Pert Pro, EMPYREAN system, PANalytical, Malvern, United Kingdom). The peaks corresponding to tetragonal zirconia, monoclinic zirconia and alumina were identified using JCPDS files. Rietveld refinement allowed the quantification of the amount of constituent phases. The starting and aged specimens were tested for biaxial flexural strength using a three-point test (piston-on-three-ball) method in a universal testing machine (Zwick/Roell Z020, Zwick Roell Group, Ulm, Germany). Each disk was placed centrally on the steel balls and the load was applied by a piston of 0.9 mm diameter. For each sub-group of ATZ composites (starting and aged), the fracture load was recorded for at least 25 specimens and the average values of biaxial flexural strength were calculated.

## 3. Results and Discussion

Table 1 shows the values of calculated theoretical density, determined final bulk density, relative density and average grain size of Al_2_O_3_ and ZrO_2_ phases. The sintering conditions used in the experiment (1450 °C, 1.5 h) led to an outstanding densification of all samples. As shown in Table 1, the achieved relative densities of ATZ composites and the reference material are superior to 99%. The influence of Al_2_O_3_ addition on the densification process of ZrO_2_-based ceramics is widely discussed in the literature; however, there are many contradictory statements on this issue [28,29]. The excellent values of relative densities reported in this paper were obtained mainly due to a very slow heating/cooling rate and good sinterability of the powders obtained by wet-chemistry techniques.

**Figure 1:**
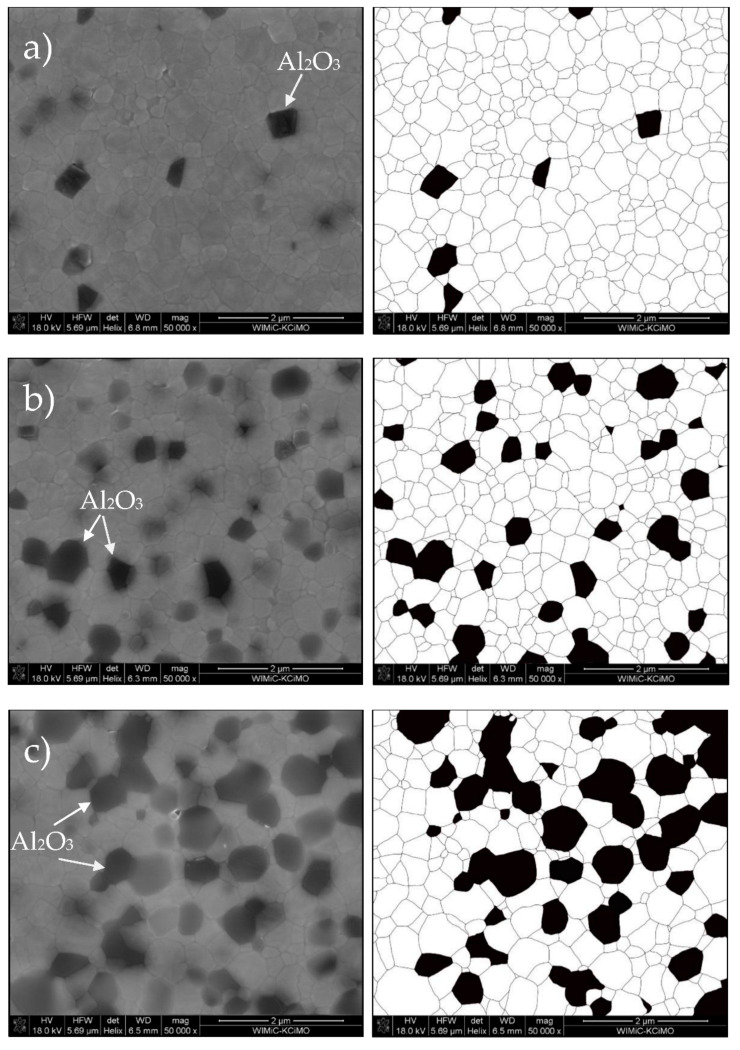

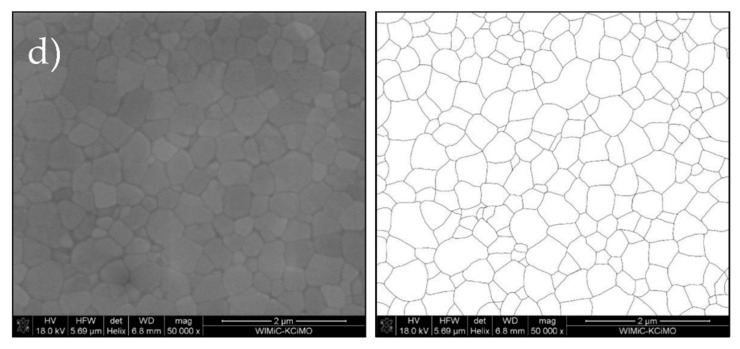
On the left side: SEM images of thermally etched (**a**) ATZ-B, (**b**) ATZ-10, (**c**) ATZ-20 composites and (**d**) 3Y-TZP reference material; on the right side: their binarized counterparts.

From Figure 1, it can be observed that all the samples had a dense, uniform and pore-free microstructure, whereas Al_2_O_3_ grains (the dark phase) were homogeneously distributed in a fine grain ZrO_2_ matrix (the brighter contrast). The applied microstructure imaging technique also captures the layer shallowly below the surface and this was clearly visible in the images as numerous shadows. Whether a grain was at the surface and was included in the microstructure analysis was determined by the course of the thermally etched boundary. The volume fractions of Al_2_O_3_ inclusions in the composites obtained as a result of the stereological analysis are almost identical to their real proportions. Slight differences, not exceeding 1%, are related to imperfect homogenization of powders, randomness of the area visible in the SEM image and also errors and limitations that result from the nature of the stereological methods themselves. The grain sizes of Al_2_O_3_ and ZrO_2_ phases were measured and calculated, and the results are reported in Table 2. The grain size of ZrO_2_ matrix was similar in every investigated ATZ composite: 0.331 ± 0.144 µm, 0.321 ± 0.145 µm and 0.335 ± 0.183 µm, respectively. Moreover, through analysis of microstructure, it was measured that the reference material, pure 3Y-TZP, had a larger average grain size, which was 0.408 ± 0.154 µm. This suggested that the addition of Al_2_O_3_ particles in the ZrO_2_ matrix acts as an inhibitor of grain growth, which agrees with Nevarez-Rascon et al. [30]. Contrary to results reported by Maji et al. [7], no significant or monotonic differences in the average grain sizes of either Al_2_O_3_ or ZrO_2_ phases were observed with an increase of Al_2_O_3_ content. No evident agglomeration of the Al_2_O_3_ phase was noticed, and the alumina grains were encapsulated in the zirconia matrix, which constrained free grain growth of Al_2_O_3_. Its average grain size in every ATZ composite was only slightly larger compared to the matrix grains; 0.484 ± 0.094 µm, 0.380 ± 0.150 µm and 0.466 ± 0.195 µm, respectively.

**Figure 2:**
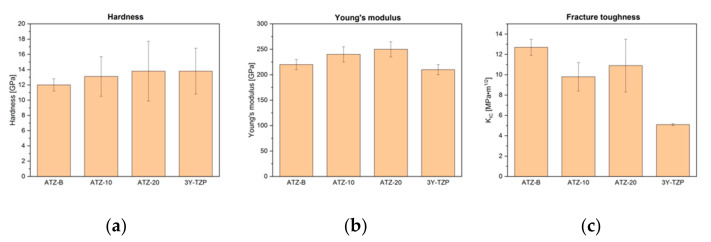
Mechanical properties of the investigated ATZ ceramics (not LTD): (**a**) hardness, (**b**) elastic modulus and (**c**) fracture toughness.

The results of the mechanical tests of Vickers hardness, Young’s modulus and fracture toughness were determined and are presented (Figure 2 and Table 3). Table 3 includes the maximum flexural strength parameter, which determined the highest result, recorded for the particular, of the best sample in a given measurement series. In the authors’ opinion, such a value provides additional information about the capabilities of the investigated materials, which are not clearly reflected in the average flexural strength, especially when the deviations of the measurement results are relatively significant. This parameter indicates the possibility of obtaining excellent ATZ composite performance under certain conditions.

The Vickers hardness was higher than 12.0 GPa, which agreed well with the early reports [3,7,9,10]. The general trend shows that the hardness values increase with increasing the Al_2_O_3_ phase content, which is commonly known to highly influence this parameter. Hence, the highest value of Vickers hardness was achieved for the ATZ-20 composition and the value was almost the same as in the 3Y-TZP reference material. It seems that Vickers hardness results are strictly associated with perfect densification and a uniform microstructure of the samples [10,30]. The former noticed a linear relationship that does exist between the Vickers hardness and relative density of ATZ composites. It should be also mentioned that such excellent results, as published here, could only be achieved for most ATZ composites when sintered at much higher temperatures, by using modern sintering methods or sintering additives [31].

Compared to the ZrO_2_ matrix (E = 210 GPa), the Al_2_O_3_ phase is characterized by almost two-fold greater elastic modulus (E = 400 GPa). This indicates that the increase in the amount of the Al_2_O_3_ would result in the increase of the Young’s modulus value, according to the rule of mixture. The obtained results confirm the assumption, as the maximum value of 250 GPa was attained for the ATZ-20 composition with the highest content of alumina phase. As expected, the lowest value of elastic modulus was measured for the reference material. The results were in good accord with the literature [32].

The highest value of fracture toughness was attained for the ATZ-B composite with the highest amount of ZrO_2_ and its K_IC_ reached 12.7 ± 0.8 MPa·m^1/2^. To the authors’ knowledge, this result is one of the best ever reported in the literature for ATZ composites (K_IC_ = 11.2 ± 0.6 MPa·m^1/2^ in [9]; K_IC_ = 6.7 ± 0.9 MPa·m^1/2^ in [7]; K_IC_~6.5 MPa·m^1/2^ in [10]; and K_IC_ = 4.8–5.2 MPa·m^1/2^ in [3]). The toughening enhancement, as compared to 3Y-TZP reference material with K_IC_ = 5.1 ± 0.1 MPa·m^1/2^, was more than double. The increase in K_IC_ value in the ATZ composites with a small amount of alumina could be explained by strong interparticle interaction due to diffusion intensification during the sintering of fine powders obtained in a presented specific way. The small addition of Al_2_O_3_ further enhanced this effect, which was also studied in [33,34]. Moreover, as was already stated in the literature [10,30,31,35,36], residual stress is created in the ATZ composites’ matrix due to the differences in thermal expansion coefficient values between the Al_2_O_3_ particles (8 × 10^−6^ K^−1^) and ZrO_2_ matrix (10.3 × 10^−6^ K^−1^). As a result, the crack deflection mechanism could be observed, which is an important factor that contributes to the enhancement of fracture toughness in ATZ composites. The analysis indicated the change in fracture mode from intergranular (present in 3Y-TZP material) to transgranular (or mix inter-transgranular) observed in the ATZ composites, which increases the energy required for crack propagation. A detailed analysis of the changes in the crack propagation mechanism is not the subject of this paper and will be published in the future.

The ATZ-10 component presents a small decrease of fracture toughness, as it contains less transformable ZrO_2_ phase. However, the ATZ-20 composite shows a slight increase of K_IC_ value in comparison with the ATZ-10 composites (however, the difference is within the measurement error). The reason for this behavior should be seen in the differences in the phase composition (amount, distribution and transformability of the ZrO_2_ tetragonal structure). In the investigated ATZ composites, the basic toughtening mechanism was the occurrence of the t-m phase transition. It is well-known that the finer the microstructure is (in the case of the presented study, a very fine microstructure was obtained, as can be seen in Figure 1 and Table 2), more intense and significant the mechanism is. An increase in Al_2_O_3_ phase content firstly reduces the contribution of this mechanism (decrease in the ZrO_2_ phase content, which is the only one to be transformed), and secondly it makes the microstructure more coarse as Al_2_O_3_ is characterized by larger grains (Table 2).

It has to be noted that there are numerous papers in the literature on mechanical properties of ATZ composites. The emerging discrepancies about the influence of Al_2_O_3_ addition in ZrO_2_ matrix and the differences in the obtained parameters values result from the diversity of starting materials and production methods. Those factors undoubtedly affect the final microstructure and phase structure of the ATZ composites and thus to a large extent determine the final properties.

Table 4 shows the quantitative phase compositions of the as-received ATZ composites and 3Y-TZP reference material estimated by XRD analysis using Rietveld refinement. From the XRD patterns with well-defined peaks, it is evident that all the samples exhibited highly crystalline structures. It was identified that ATZ-10 and ATZ-20 components were composed of the (major) tetragonal zirconia phase (t), (minor) monoclinic zirconia phase (m) and alumina phase. An Al_2_O_3_ mass content (8.3 wt.% and 15.6 wt.% for ATZ-10 and ATZ-20, respectively) represents a volume content of 11.5 vol.% for ATZ-10 and 21.0 vol.% for ATZ-20, which corresponds almost perfectly to the actual intended composition of the investigated materials. The XRD analysis of ATZ composites confirmed the absence of any secondary phase derived from the reaction of zirconia with alumina. In the 3Y-TZP reference material, only the tetragonal zirconia phase was present. The significant amount of the monoclinic zirconia phase, which was present especially in the ATZ-B composite, may be surprising. This is probably related to the nature of the powders, resulting from the unique way they were obtained, namely from a mixture of powders differing in yttria content, and to the specific rate of the heat treatment, which was optimized to achieve the best mechanical behavior of ATZ composites. Further explanation could be provided by findings reported by Bartolome [37], who stated that a certain amount of Al_2_O_3_ addition in the ZrO_2_ matrix could contribute to stabilization of the tetragonal structure in fine-grained materials during cooling from sintering (strain effects). It is suspected that a higher content of Al_2_O_3_ in ATZ-10 and ATZ-20 composites contributes to a better stabilization of the tetragonal phase. Our results appear to be in partial agreement with the conclusions presented earlier. One would expect the ATZ-20 composite (containing the most Al_2_O_3_ phase) to have the highest amount of stable tetragonal phase. However, the presence of the m-ZrO_2_ phase in ATZ-20 was much less than in the ATZ-B composite, but much higher than in the ATZ-10 component.

Table 5 reveals the results of low-temperature degradation obtained by phase quantification after XRD. Hydrothermal aging under 150 °C for 24 h led to a significant decrease in the amount of tetragonal phase (t) in all samples, the largest of which was recorded for ATZ-10 and 3Y-TZP. The composite with the highest residue of tetragonal zirconia was ATZ-B. Increasing the aging time to 48 hours caused the t-m transformation to continue, and after that time the greatest amount of the tetragonal phase remained in the ATZ-20 and 3Y-TZP materials. The further applied conditions (150 °C/72 h; 180 °C/24 h; 210 °C/24 h) proved to be very severe for the materials and an almost complete transformation of the tetragonal phase (t) into a monoclinic (m) one took place—the amount of the tetragonal zirconia dropped to only a few percent in every composite. The exception was the behavior of the 3Y-TZP reference samples, where aging over a longer period of time or at higher temperatures did not significantly affect the amount of tetragonal phase (t,) which was in the range of 17–19 wt.%, indicating high stability of the material.

**Figure 3:**
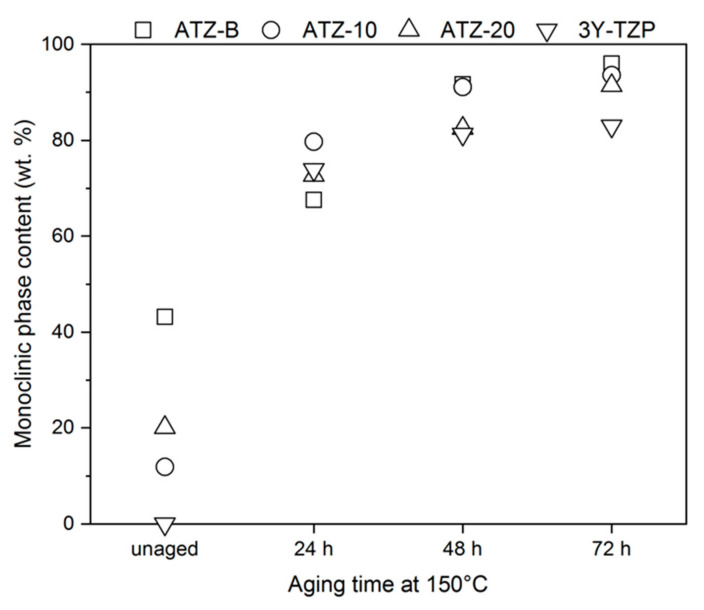
The monoclinic ZrO_2_ mass content of the investigated materials as a function of hydrothermal aging time at 150 °C.

The effects of hydrothermal aging at 150 °C are displayed in Figure 3. It can be clearly seen that the transformation rate of all the specimens was very high. Our results appear to be in agreement with Hallmann et al. [13], who found that when the average grain size exceeds 0.3 μm, the zirconia-based material exhibits high rate of hydrothermal degradation and the t-m phase transformation occurred. Moreover, according to Sequeira et al. [3], it can be assumed that the greater amount of ZrO_2_ made the materials more vulnerable to aging. The aging curves in Figure 3 also indicated that the presence of an Al_2_O_3_ phase could delayed the t-m transformation [22].

**Figure 4:**
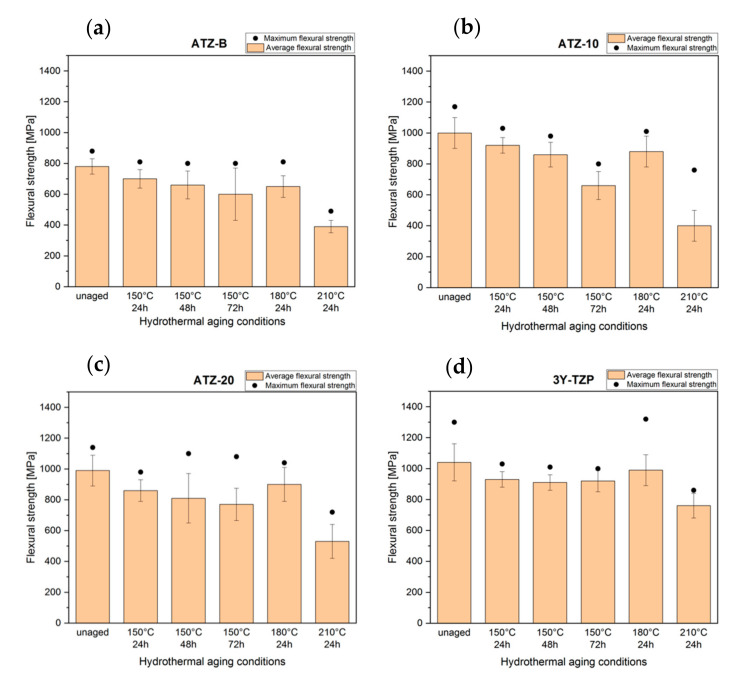
Flexural strength of (**a**) ATZ-B, (**b**) ATZ-10, (**c**) ATZ-20 composites and (**d**) 3Y-TZP reference material before and after aging. Black dots denote the maximum strength values from a given measurement series.

Biaxial flexural tests before aging reveal the high strength of 780 ± 50 MPa for ATZ-B, 1000 ± 100 MPa for ATZ-10, 990 ± 100 MPa for ATZ-20 composites and 1040 ± 120 MPa for 3Y-TZP reference material (Table 3). The values for ATZ-B, ATZ-10 and ATZ-20 were similar [9,25] or slightly higher [3,38] than those reported for corresponding compositions in the literature early. Based on literature review, the decrease in the mechanical properties as the hydrothermal degradation conditions become more severe (longer aging time and higher aging temperature) was expected. The mechanical behavior, based in this paper on the flexural strength, before and after LTD is presented in Figure 4. The 3Y-TZP reference material was the only material with stable, quite constant strength, which deteriorated only slightly under the applied hydrothermal conditions. All composites show nearly the same relative decrease in flexural strength at 150 °C in different time periods (24 h: −10.5%, −8.4% and −13.0%; 48 h: −15.0%, −14.6% and −18.4%; and 72 h: −22.6%, −34.5% and 22.5% for ATZ-B, ATZ-10 and ATZ-20, respectively). Moreover, the relative decline of flexural strength after degradation at 180 °C for 24 h was very similar to the results obtained after aging at 150 °C for 48 h (−16.2%, −12.0% and −9.9% for ATZ-B, ATZ-10 and ATZ-20, respectively). All ATZ composites were highly affected by the degradation conducted at 210 °C for 24 h and the decrease in mechanical behavior was very significant. It could be assumed that the degradation certainly expanded to the bulk of the samples.

Despite a very large increase in the amount of monoclinic zirconia phase during hydrothermal aging at 150 °C (Figure 3) and 180 °C (Table 5), a very serious decline in the flexural strength after the aging test was not detected (Figure 4). Thus, the high rate of t-m transition did not correspond and cannot be correlated directly with the deterioration of mechanical properties. This could be a clear evidence that the degradation did not expand to the bulk of the specimens and the t-m transformation occurred only on the surface, as it was observed before in [3]. After hydrothermal aging at 150 °C (24 h) and 180 °C (24 h), very good mechanical behavior of 920 MPa and 900 MPa was presented by ATZ-10 and ATZ-20 composites, respectively. However, comparison of our absolute biaxial flexural strength results with those published earlier in the literature is not possible because the earlier studies mostly dealt with aging at temperatures well below 150 °C, which is a huge difference in terms of the severity of the used conditions. The only reference is the aging test result obtained for 3Y-TZP, which showed better stability and less susceptibility to hydrothermal degradation under each of the applied conditions.

Due to the specific method of preparation, the investigated ATZ composites had a fine microstructure and grains of ZrO_2_ phase characterized by a particularly high transformability [8]. The presence of highly transformable ZrO_2_ grains gives the material high resistance to fracture toughness, while making the material very susceptible to hydrothermal aging. Investigating the possibility of improving the hydrothermal degradation resistance, an attempt was made to reduce the propensity for rapid t-m transformation of ZrO_2_ by 10 vol.% and 20 vol.% Al_2_O_3_ addition. Consequently, the K_IC_ value slightly decreased, but the flexural strength after the LTD was significantly improved. However, the behavior of traditional lower-transformable tetragonal ZrO_2_ (reference material 3Y-TZP) with coarser microstructure was still better. It should be emphasized that the ATZ-10 and ATZ-20 composites combine relatively high fracture toughness (K_IC_~10 MPa·m^1/2^) along with excellent resistance to hydrothermal degradation, only slightly worse than commercial 3Y-TZP.

## 4. Conclusions

ATZ composites were prepared from two different yttrium-containing zirconium powders synthesized by a wet chemistry method. It seems to be incontrovertible that the addition of alumina leads to the enhancement of certain mechanical behavior of ATZ composites. ZrO_2_-based materials with Al_2_O_3_ addition, depending on the composition, have very different combinations of mechanical properties that do not follow the rule of mixtures, i.e., fracture toughness or flexural strength. The investigated composites exhibited excellent biaxial flexural strength (up to 1 GPa for ATZ-10 and ATZ-20), while the addition of alumina clearly improves the crack resistance. The ATZ-B composition with minimal alumina addition has outstanding fracture toughness with K_IC_ = 12.7 MPa·m^1/2^, which was much superior to commercially available tetragonal 3Y-TZP material.

Mechanical properties that depend directly on the volume fraction of the Al_2_O_3_-ZrO_2_ components, such as hardness or Young’s modulus, change in the investigated composites according to the rule of mixtures. The more alumina in the material, the harder and the stiffer it is. Moreover, the factor that seems to have a great influence on the mechanical behavior of ATZ composites is the fine-grained, well-dense microstructure.

From evaluation of biaxial flexural strength after the low-temperature degradation (LTD) tests it was found that the aging conditions used at this work were very severe for the investigated materials. Under hydrothermal conditions at 180 °C, the extended degradation of ATZ composites took place, leading to a significant reduction of residual strength. Moreover, ATZ composites seemed to be extremely susceptible to degradation at 210 °C under hydrothermal conditions.

Even though the ATZ specimens showed high contents of monoclinic zirconia phase, a lower aging temperature of 150 °C (still quite high compared to typical potential applications’ environmental conditions) allowed the maintenance of relatively high strength. The tested ATZ composites withstood the difficult, demanding conditions (long time under hydrothermal conditions) quite well and low-temperature aging did not significantly affect the structural properties. The lowest relative flexural strength decreases (about 10–20%) were observed for 24 and 48 h aging times at 150 °C, suggesting that these materials are capable of operating under similar conditions for long periods of time without significant loss of strength.

From the density of the sintered bodies, mechanical behavior, especially outstanding fracture toughness, and LTD analysis reported in this work, the investigated ATZ composites seems to be an adequate and promising material to be considered for the fabrication of next-generation bioceramics for medical devices. Further studies are needed to increase the high temperature performance of the composites to make them suitable for use as high-performance structural ceramics.

## Figures and Tables

**Table 1:** Conditions used for autoclaving the ATZ-B, ATZ-10, ATZ-20 composites and the reference material.

Autoclaving Temperature	150 °C			180 °C	210 °C
Autoclaving time	24 h	48 h	72 h	24 h	24 h
Water vapour pressure	~5 bars	~5 bars	~5 bars	~10 bars	~20 bars

**Table 2:** Density and grain sizes of the investigated ATZ ceramics.

	ATZ-B	ATZ-10	ATZ-20	3Y-TZP	
Theoretical density [g/cm^3^]	6.01	5.81	5.61	6.10
Bulk density [g/cm^3^]	5.97	5.78	5.58	6.07
Relative density [%]	99.3	99.5	99.4	99.7
ZrO_2_ average grain size [µm]	0.331 ± 0.144	0.321 ± 0.145	0.335 ± 0.183	0.408 ± 0.154
Al_2_O_3_ average grain size [µm]	0.484 ± 0.094	0.380 ± 0.150	0.466 ± 0.195	-
Alumina content [%]	2	11	19	0

**Table 3:** Initial mechanical properties of the investigated ATZ ceramics (unaged) and 3Y-TZP reference material.

	ATZ-B	ATZ-10	ATZ-20	3Y-TZP
Hardness HV [GPa]	12.0 ± 0.8	13.1 ± 2.6	13.8 ± 3.9	13.8 ± 3.0
K_IC_ [MPa·m^1/2^]	12.7 ± 0.8	9.8 ± 1.4	10.9 ± 2.6	5.1 ± 0.1
Young’s modulus [GPa]	220 ± 10	240 ± 15	250 ± 15	210 ± 10
Flexural strength [MPa]	780 ± 50	1000 ± 100	990 ± 100	1040 ± 120
Max flexural strength [MPa]	880	1170	1140	1300

**Table 4:** Phase content of the ATZ composites and 3Y-TZP reference material in the initial state (after sintering) estimated by Rietveld refinement. m/(m + t) and t/(m + t) are the m-phase content and t-phase conten in relation to the total amount of ZrO_2_ phase, respectively.

After Sintering (Not LTD)						
	Content (wt.%)	m-ZrO_2_	$\frac{m}{m+t}\cdot 100\%$	t-ZrO_2_	$\frac{t}{m+t}\cdot 100\%$	Al_2_O_3_
Composite						
ATZ-B		42.6	43.2	55.9	56.8	1.5
ATZ-10		10.9	11.9	80.8	88.1	8.3
ATZ-20		17.0	20.1	67.4	79.9	15.6
3Y-TZP		-		100	100	-

**Table 5:** Phase content of the ATZ composites and 3Y-TZP reference material after hydrothermal treatment under selected conditions estimated by Rietveld refinement. m/(m + t) and t/(m + t) are the m-phase content and t-phase conten in relation to the total amount of ZrO_2_ phase, respectively.

Hydrothermal Aged at 150 °C/24 h						
	Content (wt.%)	m-ZrO_2_	$\frac{m}{m+t}\cdot 100\%$	t-ZrO_2_	$\frac{t}{m+t}\cdot 100\%$	Al_2_O_3_
Composite						
ATZ-B		66.6	67.6	31.9	32.4	1.5
ATZ-10		73.2	79.7	18.6	20.3	8.2
ATZ-20		61.4	72.7	23.0	27.3	15.6
3Y-TZP		73.9	73.9	26.1	26.1	-
**Hydrothermal Aged at 150 °C/48 h**						
	Content (wt.%)	m-ZrO_2_	$\frac{m}{m+t}\cdot 100\%$	t-ZrO_2_	$\frac{t}{m+t}\cdot 100\%$	Al_2_O_3_
Composite						
ATZ-B		90.3	91.7	8.2	8.3	1.5
ATZ-10		83.5	91.1	8.2	8.9	8.3
ATZ-20		69.6	82.5	14.8	17.5	15.6
3Y-TZP		81.3	81.3	18.7	18.7	-
**Hydrothermal Aged at 150 °C/72 h**						
	Content (wt.%)	m-ZrO_2_	$\frac{m}{m+t}\cdot 100\%$	t-ZrO_2_	$\frac{t}{m+t}\cdot 100\%$	Al_2_O_3_
Composite						
ATZ-B		94.6	96.0	3.9	4.0	1.5
ATZ-10		85.8	93.6	5.9	6.4	8.3
ATZ-20		77.1	91.4	7.3	8.6	15.6
3Y-TZP		83.0	83.0	17.0	17.0	-
**Hydrothermal Aged at 180 °C/24 h**						
	Content (wt.%)	m-ZrO_2_	$\frac{m}{m+t}\cdot 100\%$	t-ZrO_2_	$\frac{t}{m+t}\cdot 100\%$	Al_2_O_3_
Composite						
ATZ-B		93.5	94.9	5.0	5.1	1.5
ATZ-10		85.6	93.3	6.1	6.7	8.3
ATZ-20		77.0	91.2	7.4	8.8	15.6
3Y-TZP		81.4	81.4	18.6	18.6	-
**Hydrothermal Aged at 210 °C/24 h**						
	Content (wt.%)	m-ZrO_2_	$\frac{m}{m+t}\cdot 100\%$	t-ZrO_2_	$\frac{t}{m+t}\cdot 100\%$	Al_2_O_3_
Composite						
ATZ-B		91.9	93.3	6.6	6.7	1.5
ATZ-10		84.7	92.4	7.0	7.6	8.3
ATZ-20		78.1	92.5	6.3	7.5	15.6
3Y-TZP		81.9	81.9	18.1	18.1	-

## Data Availability

The data presented in this study are available on request from the corresponding authors.
